# An appraisal to Hans Berger by the time of his 150 ^th^ birthday: the human EEG and tales of blood flow, heat and brain waves

**DOI:** 10.1055/s-0043-1777114

**Published:** 2023-12-29

**Authors:** Marlon Wycliff Caeira, Luis Otávio Caboclo, Luciano de Paola

**Affiliations:** 1 Universidade Federal do Paraná, Hospital de Clínicas, Serviço de Epilepsia, Curitiba PR, Brazil.; 2 Hospital Israelita Albert Einstein, Departamento de Neurologia, Neurofisiologia Clínica, São Paulo SP, Brazil.

**Keywords:** Electroencephalography, History, Brain Waves, Alpha Rhythm, Eletroencefalografia, História, Ondas Encefálicas, Ritmo alfa

## Abstract

More than 100 years of research have passed by and still the human electroencephalogram (EEG) remains a puzzle to be solved. Starting from his studies on plethysmography until his theories on brain thermodynamics, Hans Berger was able to refine his method of recording cortical signs with the apparatus at his disposal in an ordinary neuropsychiatric yard towards an early account of human EEG. This review is an appraisal of his contribution to the field of modern neurophysiology.

## Resumo

Mais de 100 anos se passaram e o eletroencefalograma humano (EEG) continua sendo um enigma a ser desvendado. A partir de seus estudos sobre pletismografia até suas teorias sobre termodinâmica cerebral, Hans Berger conseguiu refinar seu método de registro da atividade elétrica cortical com os equipamentos a sua disposição em uma ala psiquiátrica comum produzindo uma descrição acurada do EEG humano. Esta revisão é um breve resumo de sua contribuição para o campo da neurofisiologia moderna.

## INTRODUCTION

**Figure 1:**
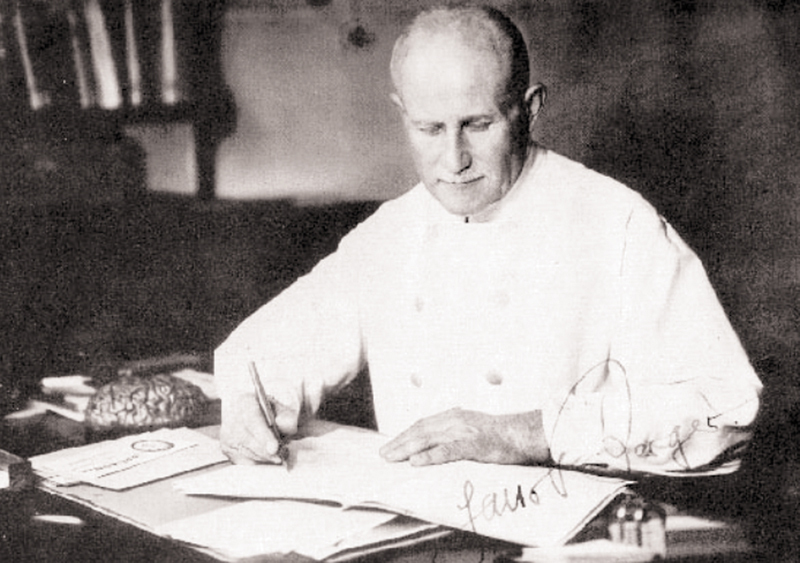
Hans Berger (1873-1941) as portraited in 1930. By the time this photograph was taken, Berger had already recorded dozens of EEGs and started to publish his reports on human EEG. 1

Some 150 years ago, on May 21st, 1873, in the town of Coburg, south Germany, Hans Berger (1873-1941) was born ( Figure 1 ). Son to Paul Friedrich Berger, chief physician of the regional asylum, and grandson by mother side of the famous Franconian poet Friedrich Rückert. Although inspired by this academic ancestry, young Berger did not show at first any particular interest in pursuing a career in Medicine and at the age of 18 enrolled at the University of Berlin to study mathematics and astronomy. 1

There he stood for half a year before quitting his studies and enlisting the army to serve the Kaiser in Würzburg. In 1892, while pulling some artillery material, he fell from a horse in an accident that almost took his life. Rumor has it that many miles away in his hometown, Hans̀ older sister was taken, at that very moment, by a feeling her brother has been in grave peril, leading her father to send a telegram to check for Berger̀s health. 2 3 From that moment forward, a stark and unwavering resolution lodged in his mind to determine the means of what he termed telepathy.

To pursue this endeavor, Berger resumed his academic life focusing on Medicine. He studied in Würzburg, Berlin, Munich, Jena, and Kiel and finally achieved his degree in 1897. 4 At this time point, he was invited by Otto Binswanger to join him at Friedrich Schiller University Hospital of Jena as an assistant. 3 5 Berger had a brilliant academic career at Jena, where he completed his *Habilitation* (i.e., qualification as an university lecturer) in 1901 with a monograph concerning cerebral blood flow variations under influence of different substances, 3 6 was promoted to chief physician of the University Clinic at 1912 1 and took Binswanger place as Universitỳs Chair and Hospital Director between 1919-1938. 4 In the meantime, he also became Rector of the Friedrich Schiller University in 1927. 4

The basic principle that guided Berger̀s thinking is conservation of energy. Borrowed from the newborn Thermodynamics it was explored in the field of psychophysiology by scholars like the Austrian neuropsychiatrist Theodor Meynert and the Danish experimental psychologist Alfred Lehmann. The latter had produced a deep impression on Berger̀s thinking. Lehmann proposed that brain energy was constant and a byproduct of the summation of three basic forms of energy: heat, electricity, and what Lehmann called “P-energy” or psychic energy, which was ultimately related to mental work and the precise feature that distinguished the organism with a soul from one without. 7 That was exactly the theoretical support Berger needed to start his investigations on a biophysical model for the transformation of energy by the brain. 2

Berger̀s starting point was the most obvious surrogate of brain metabolism: blood supply. To Meynert̀s psychodynamic theory, when a certain part of the brain is activated by a thought or emotion resulting in energy consumption, an equal amount of energy should spontaneously disappear from another region in order to fulfil the principle of conservation of energy. This redistribution of energy would be led by vasomotor centers is the brain, producing *hyperemia* at the active region of the cortex and vasoconstriction in its inhibited counterpart. 1

Berger̀s studies on cerebral blood flow were particularly influenced by his first position at Friedrich Schiller University. He was pointed as an assistant to Theodor Ziehen, chief physician of the University Clinic during Berger̀s early academic years. Ziehen had dedicated his career to attempt to find new ways to diagnose psychiatric conditions, introducing sphygmography (i.e., the recording of pulse curves) at Jena to study mental illnesses. As Zieheǹs first results turned negative, he asked Hans Berger and Korbinian Brodmann to use another graphic method to study the process of mental disease. The proposed technique was plethysmography (i.e., the record of volume changes in body parts) which would work as a “psychoscope” in Lehmanǹs own words “when all mental states and conditions were examined”. 3

Using plethysmography, Berger was able to investigate cerebral blood flow variations in patients with acquired skull defects (e.g., craniotomies, gunshots, infections) under different mental states or environmental stimuli and while performing non-motor tasks, such as arithmetic. The method used by Berger in this effort was exquisitely elegant and did not include invasive measurements. He used instead an adaptation of the technique employed by the Italian physiologist Angelo Mosso. Berger filled a cap made of gutta-percha with water and attached its edges to the borders of the skull defects of his patients while performing the experiments. The apparatus was then connected to a Mary-type tambour to obtain the graphic records of volume changes in intracranial content. 1 To eliminate pulse artifacts, he also measured arm volume changes showing a methodological rigor that would later prove to be crucial for recording brain electrical activity. 1 2

**Figure 2:**
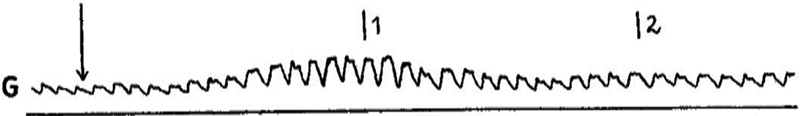
One of Berger̀s plethysmographic records. The alteration in the baseline was attributed to the feeling of anger. This tracing was reproduced by Sigmund Exner in his review on central nervous system physiology of Nathan Zuntz and Adolf Loweỳs *Lehrbuch der Physiologie* . 8

While Brodmann abandoned this line of research to dedicate to his cytoarchitectonic analysis of the cortex soon after, Berger achieved a lot of positive results and published two monographs and an atlas detailing the variations in cerebral blood flow under diverse psychic phenomena such as pain, pleasure, touch, hearing and mental arithmetic ( Figure 2 ). 3 8 He was even able to demonstrate by close analysis of his tracings that small cortical vessels were those responsible for variations in blood flow to the cerebral cortex. 1 In fact, Berger became an authority in the field of psychic physiology at the beginning of the 20 ^th^ century figuring alongside Mosso and Lehmann in the section on the central nervous system in one of the most widespread physiology textbooks of the time. 3 8

Nonetheless, plethysmography failed as a model for explaining energy transformation by mental processes, Berger̀s utmost goal. Furthermore, the method was plenished with technical and theoretical pitfalls, making it difficult to obtain clear and trustful records. 1 9

Alongside his cerebral blood flow studies, in 1902 and 1907 Berger tried to replicate the work of Richard Caton which consisted of recording electrical currents directly from the surface of dog̀s brains. Similar experiments had already been conducted by Danilevsky, Beck, and Cybulski in Eastern Europe and were proven successful. 10 Berger used the standard apparatus at disposal in physiology laboratories at that time, a Lippmann capillary electrometer, to take his records. At the first attempt in 1902, he failed in four of five registries and got only a feeble signal in the remainder. 1 10 As none of his efforts has proven fruitful so far, Berger moved to study brain temperature. 2 9

Again, Angelo Mosso comes in Berger̀s path as a source of inspiration. While analyzing temperature fluctuations, Mosso concluded that brain temperature fluctuates regardless of blood temperature and argued that this difference should be related to the chemical energy released by brain metabolic activity itself. 11 The second element of Berger̀s rationale in studying brain temperature was Max Verworn's concept of *biotonus* . According to Verworn, who was a professor of Physiology at Jena, normal tissular function rests on the chemical balance between assimilation (i.e., the process of nutrients being taken up and built into proteins) and dissimilation (i.e., the process of breakdown of proteins to support living cells). 1 Berger hypothesized that when the process of dissimilation achieved a certain threshold in the cortex it would exceed the capacity of the tissue to convert chemical energy into heat and electricity and the remainder would be released as Lehmanǹs “P-energy”. If he could measure the amount of energy expended as heat, then he could estimate the amount of energy available for mental processes. 1 9 Taking advantage of the technique of brain puncture to localize tumors intraoperatively, Berger started to use mercury precision thermometers to measure temperature changes in the brain under several experimental conditions. 9

After performing numerous measures in different mental states (e.g., chloroform narcosis, awakening from anesthesia, performing mental calculations), he was able to estimate the amount of energy needed to raise brain temperature by one degree centigrade (i.e., 348kcal) and then estimate the upper limit of energy available for mental work (i.e., roughly 3 J/min or 0.05 W, the same amount of energy required to power 500 times a modern calculator). 1 In 1910, he published his results in a monograph entitled *Investigations on Brain Temperature* . 9

Despite his successful thermometric analysis of the brain, Berger was still unsatisfied and saw his psychodynamic model as an incomplete theory for psychic energy. 9 He was able to measure the energy released by the cortex as heat, but he could only estimate the energy available for mental work based on a feeble extrapolation of Adolf Fick̀s studies of muscle physiology about the proportion of energy transformed by living tissues as heat and as other forms of work (according to Berger̀s calculations 40% and 60% of brain metabolic supply, respectively). 1 To grasp the enigma, there were still two gaps to be filled: the fraction of the brain's energy released as electricity and finally the remaining energy available as psychic energy.

**Figure 3:**
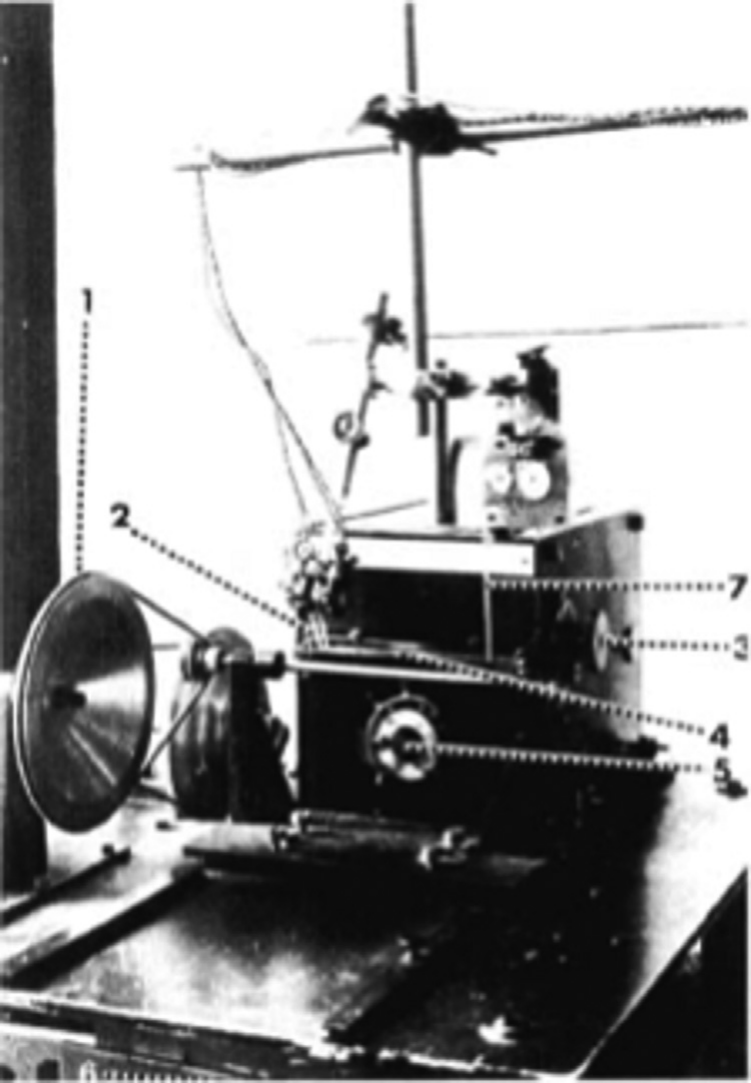
Hans Berger̀s Edelmann string galvanometer. 1. Crank, 2. Marker fibers, 3. On/off switch, 4. Lens, 5. Diaphragm, 6. Paper box (at the right bottom) and 7. Turning fork. 13

Frustrated with the fruits of his efforts so far and after publishing his monograph on brain temperature, he resumed his experiments on brain surface electrical currents evoked by sensory stimuli using an Einthoven string galvanometer and later a small Edelmann string galvanometer used in the clinic to take EKGs ( Figure 3 ). 10 12 With the latter, he took photographic records 1-3 min in length over tapes measuring 5-6 cm of silver bromide paper which needed to be developed in a darkroom after tracing in order to be read. 10 Again, his results were only marginal, and he abandoned this enterprise in the meantime. 1

At the beginning of the 1910s, recurrent episodes of depression, his marriage to the baroness Ursula von Bülow – a technician in the Psychiatric Clinic – in 1911, his appointment as chief physician of the University Clinics in 1912, and the outbreak of World War I in 1914 left Berger with little to no time to dedicate to his research on cerebral energetics. During WWI, he was deployed to serve as a neuropsychiatrist at the western front in Rethel, which gave him time to study English, and human sciences (particularly philosophy) and to make plans for his research in the after-war. 1

Berger resumed his work on human electroencephalogram (EEG) only in 1924. 10 At that time, cortical stimulation was already established and a routine to eminent physiologists and clinicians, like Harvey Cushing and Charles Sherrington, and the problem of brain electrical currents came to Berger̀s attention again. His new hypothesis was that when stimulated by small electrical currents, different areas of the cortex would show distinctive degrees of dissimilation resulting in psychological effects depending on which area was stimulated, either motor or sensory areas. 1

His first subject was a 17-year-old boy who had undergone a frontoparietal craniotomy leaving a cranial defect over the central sulcus and adjacent gyri. His experiment consisted of stimulating the cortex to induce the dissimilation process while using the same set of electrodes to record the electrical current that would rise from the resulting breakdown of the organic compounds. He used a set of du Bois-Reymond clay electrodes attached to a small Edelmann string galvanometer in a very similar way he used in his animal experiments in the early 1900s. 1 10 In July 6 ^th^ , 1924 a feeble current was observed when the electrodes were placed 4 cm apart just around a scar over the scalp after several days and many adjustments in the equipment. He needed a 5.200-ohm platinum thread or a 3.200-ohm quartz thread to observe any movement, however, a 100-times magnification was needed to perform a record 5 10 and any further attempt to increase the sensitivity of the galvanometer often resulted in damage to its delicate components or electrical noise. 1

These technical drawbacks pointed to the need for a more sensitive but reliable instrument for recording. At first, he used a larger Edelmann string galvanometer with a sensitivity of 1 mV/cm and a frequency response of 200 Hz along with a set of brush nonpolarizable electrodes. The records were still difficult to obtain, even at exposed surfaces of the cortex, since the electrodes he was using were producing resistances as high as 44.000 ohms. 10

**Figure 4:**
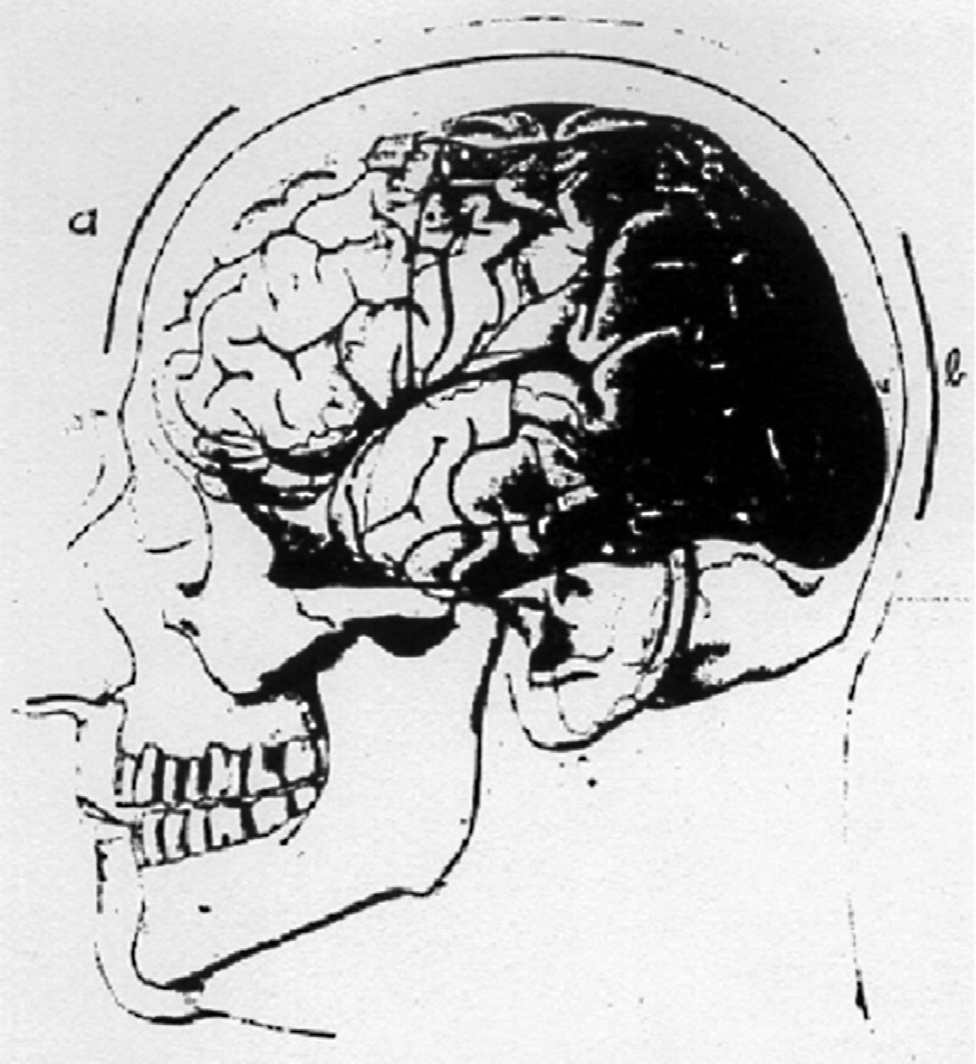
Berger̀s schematic illustration of lead electrodes disposition over the forehead and occipital region, just above the inion. The attaching material used to fix the electrodes is not shown. 13 14

In 1926, Berger started to use a Siemens double-coil galvanometer which provided him with a sensitivity of 130 μV/cm and allowed records with surface low impedance electrodes. 2 The adaptation of his technique was followed with improvements in the electrodes in order to reduce resistance. First, he abandoned clay and zinc sulfate nonpolarizable electrodes in favor of polarizable metal electrodes made of platinum, silver, or lead. Second, he increased the concentration of electrolytes in the electrodes̀ pads using copper plates and a 20% sodium chloride solution. With these measures, he could achieve resistances as low as 240 ohms. 10 His preferred montage for intact scalp records was an anteroposterior bipolar longitudinal montage linking two lead plates attached at the forehead and at the mid-occipital region by rubber bands or tape in order to have mechanical stability ( Figure 4 ). 2 13

Berger had a brother who was an engineer at the Carl Zeiss Company and this connection proved crucial at the time of his EEG scientific production, as Berger received grants and assistance from the Carl Zeiss Foundation to acquire his sensitive record equipment, especially the Siemens oscillators and double-coil galvanometers. 10

Always skeptical of his own research and afraid of unintended appropriation of his ideas, Berger worked in the evening between 5:00 and 8:00 p.m. in almost complete isolation and perfected his method for five years before publishing his first report on human EEG in 1929. 5 14 He performed 73 records of his son Klaus, whose hair was cut as short as possible before the EEGs were taken, 56 traces from himself using needle electrodes placed under the skin and hundreds of EEGs from patients with and without skull defects and also from healthy subjects. 1 10 For his efforts, Berger used galvanometers of 2 separated channels maximum and never had had access to any equipment with an ink-writing system. He possessed such a stark scientific rigor that all his records were provided with a 1/10s time scale and controls in order to prove the rhythms were truly brain-derived and not noise produced by EKG or muscle activity. 2

**Figure 5:**
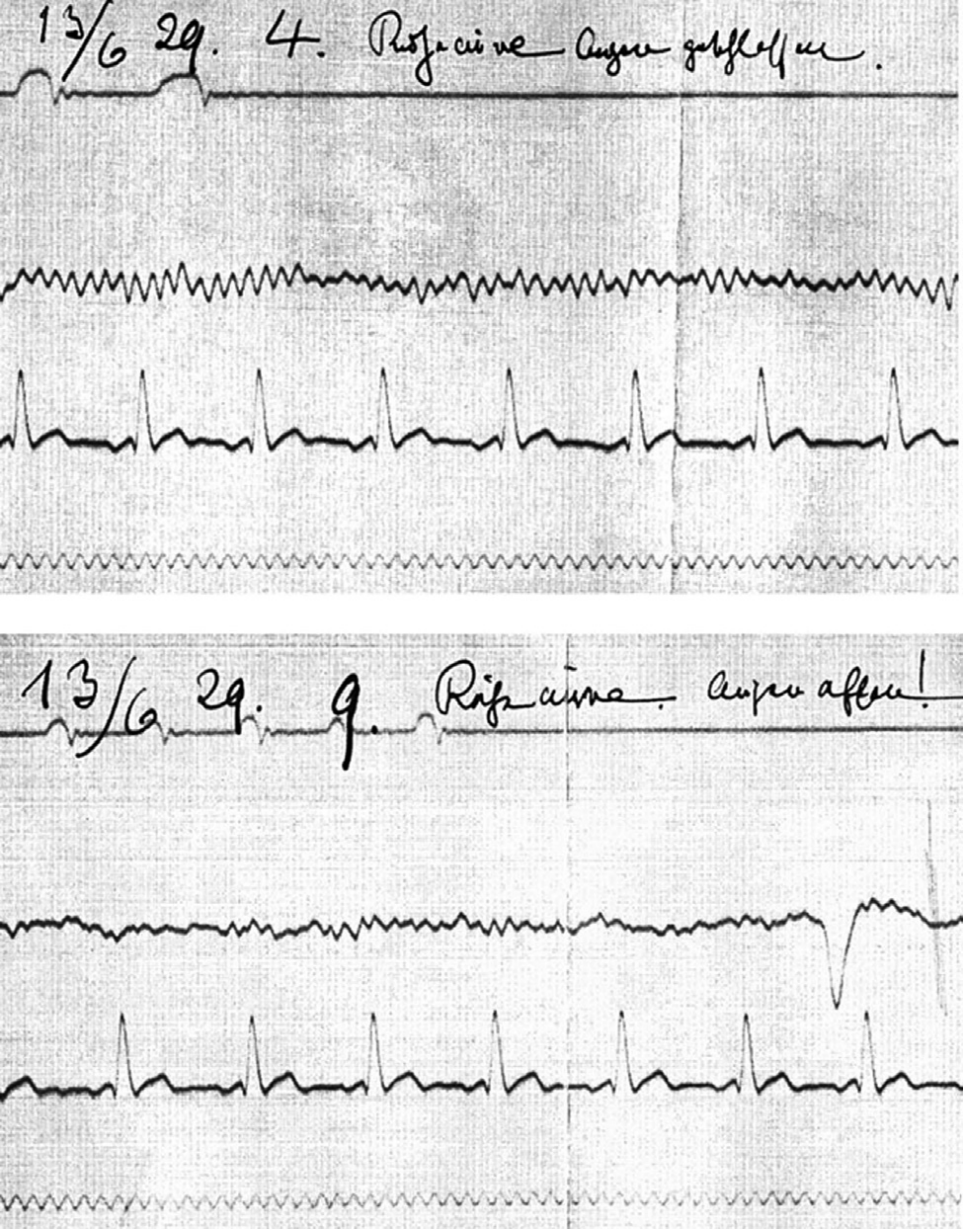
Berger experiment on the blocking of alpha rhythm by eye opening. At the upper panel, the individual lies with eyes closed and at the lower panel, with eyes opened. In order of appearance: 1) EEG, 2) EKG and 3) 1/10 s markings. 15

After 1929, Berger started to publish his reports in a series of 14 papers, mainly in German psychiatry journals instead of physiology ones ( Figure 5 ). 15 By 1931, he had gathered 1133 records from 76 people establishing a normalization for alpha and beta waves in terms of frequency and amplitude. 10 At this time, he used a Siemens amplifier/oscillator so the traces could be taken irrespective of electrode resistance. Also, he had changed from foil or sheet electrodes to chloride silver needle electrodes inserted at the periosteum under local anesthetics to reduce artifacts. The records were taken in 12-cm width paper, running at an average of 3 cm/s and with a maximum length of 7,5 meters. 10

In 1938 he compiled his work in a monograph published in the “Nova Acta Leopoldina”. 15 16 This monograph comprised a description of normal EEG of the adult with his alpha and beta waves, the blocking of alpha waves in response to eye-opening or sensory stimuli, named brain waves in terms of their frequency (i.e. alpha, beta, theta and delta waves), the EEG across age groups (newborns, children, elderly), the isoelectric EEG in brain death, the effect of narcotics and hyperventilation on EEG and EEG changes in different pathological states (intracranial hemorrhage, dementia, neoplasms, interictal epileptiform activity, *petit mal* seizures and the background attenuation following a *grand mal* seizure). 15

Some reasons could explain the skepticism Berger̀s publications received by the scientific community of his time. First, in Jasper̀s words, it seemed unlikely that in the chaotic multitude of action potentials of brain cells running in all directions and with multiple connections any rhythm or orderly activity could be recorded from the scalp. 1 Second, contrasting many of his peers in Neurologỳs Pantheon like Broca and Brodmann, Berger did not think his method was as a matter of location of functional regions. Though he was no stranger to these matters, his approach was fashioned to understand the way the brain *works* either in healthy or pathological states – to unveil the paradox of the mind and the brain it lies in, as stated by his inaugural lecture as Director of the Psychiatric Clinic: “Brain and Soul”. 1 12 17 This apparent disconnection of the EEG and cortical localization was unacceptable to German neurophysiologists, especially the hegemonic Berlin-Buch group, who saw Berger as naïve, misguided and provided with no technical support. 1

Berger̀s isolation at Jena also played a role in this matter, by means of his seclusive personality, fear his ideas were stolen, his conservative political views in a transitional Germany, Binswanger̀s heavy lobby to favor Berger as heir to his position as Director of the University Clinics in place of external more suitable candidates and Binswanger own discredit on Berger̀s clinical skills (he lend his private practice to Strohmayer instead of Berger). 1

Only in 1934, five years after his first report, Berger received some acknowledgment, as 1932̀s Nobel Prize winner Douglas Adrian and his engineer Brian Matthews reproduced EEG recordings, including the alpha rhythm – which Adrian termed *Berger Rhythm* – and its blocking by eye-opening. 2 17 Their initial intention was to prove Berger̀s records were related to noise and nothing but artifactual, but after clear reproducible traces, they promptly published a report. 5 In what seems to be an ironic reduction of physiological EEG phenomena, Adrian and Matthews even compared Adriaǹs own alpha rhythm to a water-beetlès ganglionic activity (including the alpha-blocking by eye-opening/visual stimuli) in their original report. 18

As Berger published his papers in non-physiology journals and only in German, Adrian and Matthews̀ publication in the English language in a physiology journal was crucial for EEG diffusion worldwide, and in 1937 Berger presided over a symposium with Adrian on the subject. 1 5 The EEG would become a widespread neurological tool, to be perfected by names like the Gibbses, Lennox, and Jasper in the Americas and Gastaut in France. 4

In 1935, Berger was forced to abandon his research on EEG by the Nazi party, but he kept his publications with the notes and the material gathered so far. 2 In 1938, he wasǹt reappointed by the Nazy regime to his position as Hospital Director, eventually leaving Berger at scientific ostracism. 4 On June 1 ^st^ , 1941, during a severe episode of depression and after a long process of decay due to heart failure, Berger committed suicide by hanging. 1 3

Though a matter of debate, canonical medical literature points to two dates for Berger to receive the Nobel Prize, first in 1936 and later in 1949. The Prize was not granted in 1936 since Hitler forbade all German scholars from receiving it after the German-Jewish Journalist Carl von Ossietzky was granted the Nobel Peace Prize in 1935. In 1949, Berger was long dead and so the Prize was not granted either. 19

At Berger's 150-year anniversary, some hundred years after his first report on human EEG, the basic principles of his technique with string galvanometers are still the same we apply today in modern digital records. The contributions of Gibbs, Lennox, Gastaut, Jasper, Penfield, and others were only possible because of Berger̀s perseverance even when his results were negative or dumped into discredit among his peers. Ahead of his time, Berger̀s approach to brain energetics is astonishingly akin to the concepts used in modern functional imaging through MRI and PET scans. In a time when we thrive with artificial intelligence and sail the oceans of neuroscience searching so eagerly for a perfect interface between the human brain and machines, it is worth looking back to this pioneer, whose invention unveiled the secrets of brain waves to neurologists and neurophysiologists.
